# Supplementary material for: Proximal Active Optical Sensing Operational Improvement for Research Using the CropCircle ACS-470, Implications for Measurement of Normalized Difference Vegetation Index (NDVI)
Source: Sensors (Basel). 2023 May 24;23(11):5044. doi: 10.3390/s23115044 (PMC10255205; doi:10.3390/s23115044)
Supplement: Supplementary file 1 [file sensors-23-05044-s001.zip › File S1 ANOVA.pdf]

Statistical analysis of data included in Figure 10

Analysis of individual sensor detector change in white panel reflectance measured before and after 62 field collection events across four years that show individual detectors behave differently. Data includes all three detectors of all sensors from the "Wolverine" field collections taken 2018-2021. Results produced using JMP, build 15.2.0 SAS Institute Inc. Cary North Carolina 27513

Oneway Analysis of signal change (median_diff) by individual (detector)

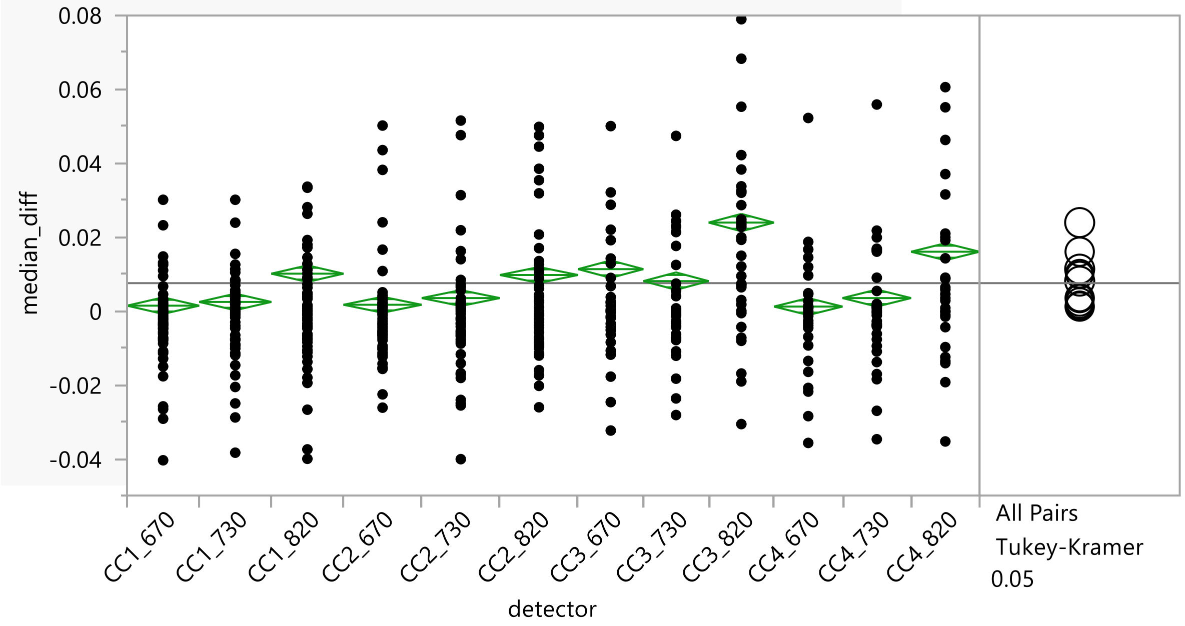

Oneway Anova
Summary of Fit

Rsquare	0.137102
Adj Rsquare	0.133226
Root Mean Square Error	0.016507
Mean of Response	0.007527
Observations (or Sum Wgts)	2460.771

Analysis of Variance

Source	DF	Sum of Squares	Mean Square	F Ratio	Prob > F
detector	11	0.10601629	0.009638	35.3703	<.0001*
Error	2449	0.66725058	0.000272		
C. Total	2460	0.77326687			

Means for Oneway Anova

Level	Number	Mean	Std Error	Lower 95%	Upper 95%
CC1_670	208.197	0.001426	0.00114	-0.0008	0.00367
CC1_730	208.197	0.002437	0.00114	0.00019	0.00468
CC1_820	208.197	0.010089	0.00114	0.0078	0.01233
CC2_670	225.77	0.001671	0.00110	-0.0005	0.00383
CC2_730	225.77	0.003481	0.00110	0.0013	0.00564
CC2_820	225.77	0.009731	0.00110	0.0076	0.01188
CC3_670	188.45	0.011316	0.00120	0.0090	0.01367
CC3_730	188.45	0.008075	0.00120	0.0057	0.01043
CC3_820	188.45	0.023928	0.00120	0.0216	0.02629
CC4_670	197.84	0.001179	0.00117	-0.0011	0.00348
CC4_730	197.84	0.003482	0.00117	0.0012	0.00578
CC4_820	197.84	0.016033	0.00117	0.0137	0.01833

Std Error uses a pooled estimate of error variance

Means Comparisons

Comparisons for all pairs using Tukey-Kramer HSD

Confidence Quantile

q*	Alpha
3.27120	0.05

HSD Threshold Matrix

Abs(Dif)-HSD

	CC3_820	CC4_820	CC3_670	CC1_820	CC2_820	CC3_730	CC4_730	CC2_730	CC1_730	CC2_670	CC1_670	CC4_670
CC3_820	-0.00556	0.00240	0.00705	0.00841	0.00887	0.01029	0.01495	0.01512	0.01606	0.01693	0.01707	0.01725
CC4_820	0.00240	-0.00543	-0.00078	0.00058	0.00104	0.00246	0.00712	0.00729	0.00823	0.00910	0.00925	0.00942
CC3_670	0.00705	-0.00078	-0.00556	-0.00420	-0.00374	-0.00232	0.00234	0.00251	0.00345	0.00432	0.00446	0.00464
CC1_820	0.00841	0.00058	-0.00420	-0.00529	-0.00483	-0.00341	0.00125	0.00142	0.00236	0.00323	0.00337	0.00355
CC2_820	0.00887	0.00104	-0.00374	-0.00483	-0.00508	-0.00367	0.00099	0.00117	0.00210	0.00298	0.00312	0.00329
CC3_730	0.01029	0.00246	-0.00232	-0.00341	-0.00367	-0.00556	-0.00090	-0.00073	0.00021	0.00108	0.00122	0.00140
CC4_730	0.01495	0.00712	0.00234	0.00125	0.00099	-0.00090	-0.00543	-0.00526	-0.00432	-0.00345	-0.00331	-0.00313
CC2_730	0.01512	0.00729	0.00251	0.00142	0.00117	-0.00073	-0.00526	-0.00508	-0.00414	-0.00327	-0.00313	-0.00296
CC1_730	0.01606	0.00823	0.00345	0.00236	0.00210	0.00021	-0.00432	-0.00414	-0.00529	-0.00442	-0.00428	-0.00410
CC2_670	0.01693	0.00910	0.00432	0.00323	0.00298	0.00108	-0.00345	-0.00327	-0.00442	-0.00508	-0.00494	-0.00477
CC1_670	0.01707	0.00925	0.00446	0.00337	0.00312	0.00122	-0.00331	-0.00313	-0.00428	-0.00494	-0.00529	-0.00511
CC4_670	0.01725	0.00942	0.00464	0.00355	0.00329	0.00140	-0.00313	-0.00296	-0.00410	-0.00477	-0.00511	-0.00543

Positive values show pairs of means that are significantly different.

Connecting Letters Report

Level						Mean
CC3_820	A					0.02392772
CC4_820		B				0.01603268
CC3_670		B	C			0.01131588
CC1_820			C			0.01008920
CC2_820			C			0.00973053
CC3_730			C	D		0.00807457
CC4_730				D	E	0.00348188

Level			Mean
CC2_730	D	E	0.00348110
CC1_730		E	0.00243740
CC2_670		E	0.00167112
CC1_670		E	0.00142613
CC4_670		E	0.00117947

Levels not connected by same letter are significantly different.

Ordered Differences Report

Level	- Level	Difference	Std Err Dif	Lower CL	Upper CL	p-Value
CC3_820	CC4_670	0.0227482	0.0016802	0.017252	0.0282446	<.0001*
CC3_820	CC1_670	0.0225016	0.0016597	0.017072	0.0279309	<.0001*
CC3_820	CC2_670	0.0222566	0.0016288	0.016929	0.0275846	<.0001*
CC3_820	CC1_730	0.0214903	0.0016597	0.016061	0.0269196	<.0001*
CC3_820	CC2_730	0.0204466	0.0016288	0.015119	0.0257746	<.0001*
CC3_820	CC4_730	0.0204458	0.0016802	0.014949	0.0259422	<.0001*
CC3_820	CC3_730	0.0158531	0.0017005	0.010290	0.0214160	<.0001*
CC4_820	CC4_670	0.0148532	0.0016597	0.009424	0.0202824	<.0001*
CC4_820	CC1_670	0.0146065	0.0016389	0.009245	0.0199678	<.0001*
CC4_820	CC2_670	0.0143616	0.0016075	0.009103	0.0196202	<.0001*
CC3_820	CC2_820	0.0141972	0.0016288	0.008869	0.0195251	<.0001*
CC3_820	CC1_820	0.0138385	0.0016597	0.008409	0.0192678	<.0001*
CC4_820	CC1_730	0.0135953	0.0016389	0.008234	0.0189565	<.0001*
CC3_820	CC3_670	0.0126118	0.0017005	0.007049	0.0181746	<.0001*
CC4_820	CC2_730	0.0125516	0.0016075	0.007293	0.0178102	<.0001*
CC4_820	CC4_730	0.0125508	0.0016597	0.007122	0.0179800	<.0001*
CC3_670	CC4_670	0.0101364	0.0016802	0.004640	0.0156328	<.0001*
CC3_670	CC1_670	0.0098898	0.0016597	0.004460	0.0153191	<.0001*
CC3_670	CC2_670	0.0096448	0.0016288	0.004317	0.0149727	<.0001*
CC1_820	CC4_670	0.0089097	0.0016389	0.003548	0.0142710	<.0001*
CC3_670	CC1_730	0.0088785	0.0016597	0.003449	0.0143078	<.0001*
CC1_820	CC1_670	0.0086631	0.0016179	0.003371	0.0139555	<.0001*
CC2_820	CC4_670	0.0085511	0.0016075	0.003292	0.0138097	<.0001*
CC1_820	CC2_670	0.0084181	0.0015861	0.003230	0.0136065	<.0001*
CC2_820	CC1_670	0.0083044	0.0015861	0.003116	0.0134928	<.0001*
CC2_820	CC2_670	0.0080594	0.0015536	0.002977	0.0131417	<.0001*
CC4_820	CC3_730	0.0079581	0.0016802	0.002462	0.0134545	0.0001*
CC3_820	CC4_820	0.0078950	0.0016802	0.002399	0.0133914	0.0002*
CC3_670	CC2_730	0.0078348	0.0016288	0.002507	0.0131628	0.0001*
CC3_670	CC4_730	0.0078340	0.0016802	0.002338	0.0133304	0.0002*
CC1_820	CC1_730	0.0076518	0.0016179	0.002359	0.0129442	0.0002*
CC2_820	CC1_730	0.0072931	0.0015861	0.002105	0.0124816	0.0003*
CC3_730	CC4_670	0.0068951	0.0016802	0.001399	0.0123915	0.0025*
CC3_730	CC1_670	0.0066484	0.0016597	0.001219	0.0120777	0.0037*
CC1_820	CC2_730	0.0066081	0.0015861	0.001420	0.0117965	0.0019*
CC1_820	CC4_730	0.0066073	0.0016389	0.001246	0.0119686	0.0033*
CC3_730	CC2_670	0.0064034	0.0016288	0.001075	0.0117314	0.0049*
CC4_820	CC2_820	0.0063021	0.0016075	0.001044	0.0115607	0.0052*
CC2_820	CC2_730	0.0062494	0.0015536	0.001167	0.0113317	0.0034*
CC2_820	CC4_730	0.0062487	0.0016075	0.000990	0.0115073	0.0059*

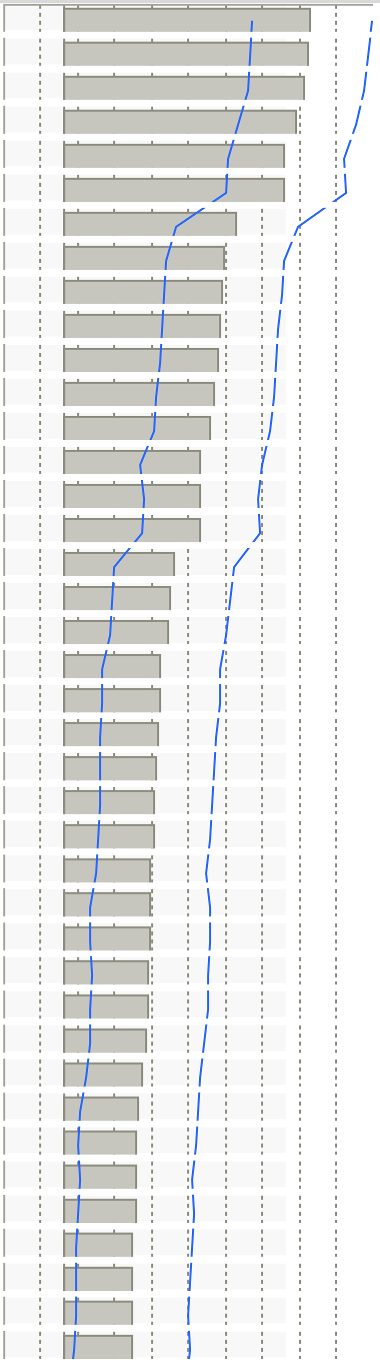

Level	- Level	Difference	Std Err Dif	Lower CL	Upper CL	p-Value
CC4_820	CC1_820	0.0059435	0.0016389	0.000582	0.0113047	0.0154*
CC3_730	CC1_730	0.0056372	0.0016597	0.000208	0.0110665	0.0337*
CC4_820	CC3_670	0.0047168	0.0016802	-0.000780	0.0102132	0.1774
CC3_730	CC2_730	0.0045935	0.0016288	-0.000734	0.0099214	0.1720
CC3_730	CC4_730	0.0045927	0.0016802	-0.000904	0.0100891	0.2104
CC3_670	CC3_730	0.0032413	0.0017005	-0.002321	0.0088041	0.7556
CC4_730	CC4_670	0.0023024	0.0016597	-0.003127	0.0077316	0.9661
CC2_730	CC4_670	0.0023016	0.0016075	-0.002957	0.0075602	0.9573
CC4_730	CC1_670	0.0020557	0.0016389	-0.003305	0.0074170	0.9843
CC2_730	CC1_670	0.0020550	0.0015861	-0.003133	0.0072434	0.9798
CC1_820	CC3_730	0.0020146	0.0016597	-0.003415	0.0074439	0.9880
CC4_730	CC2_670	0.0018108	0.0016075	-0.003448	0.0070694	0.9935
CC2_730	CC2_670	0.0018100	0.0015536	-0.003272	0.0068923	0.9914
CC2_820	CC3_730	0.0016560	0.0016288	-0.003672	0.0069839	0.9974
CC3_670	CC2_820	0.0015853	0.0016288	-0.003743	0.0069133	0.9982
CC1_730	CC4_670	0.0012579	0.0016389	-0.004103	0.0066192	0.9998
CC3_670	CC1_820	0.0012267	0.0016597	-0.004203	0.0066560	0.9999
CC4_730	CC1_730	0.0010445	0.0016389	-0.004317	0.0064057	1.0000
CC2_730	CC1_730	0.0010437	0.0015861	-0.004145	0.0062321	1.0000
CC1_730	CC1_670	0.0010113	0.0016179	-0.004281	0.0063037	1.0000
CC1_730	CC2_670	0.0007663	0.0015861	-0.004422	0.0059547	1.0000
CC2_670	CC4_670	0.0004916	0.0016075	-0.004767	0.0057502	1.0000
CC1_820	CC2_820	0.0003587	0.0015861	-0.004830	0.0055471	1.0000
CC1_670	CC4_670	0.0002467	0.0016389	-0.005115	0.0056079	1.0000
CC2_670	CC1_670	0.0002450	0.0015861	-0.004943	0.0054334	1.0000
CC4_730	CC2_730	7.8096e-7	0.0016075	-0.005258	0.0052594	1.0000

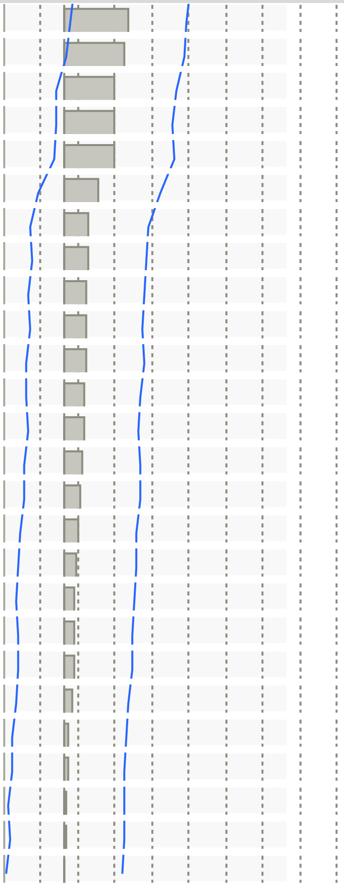

Freq temp_diff
No Weight Rows 141
