# Supplementary material for: Proximal Active Optical Sensing Operational Improvement for Research Using the CropCircle ACS-470, Implications for Measurement of Normalized Difference Vegetation Index (NDVI)
Source: Sensors (Basel). 2023 May 24;23(11):5044. doi: 10.3390/s23115044 (PMC10255205; doi:10.3390/s23115044)
Supplement: Supplementary file 1 [file sensors-23-05044-s001.zip › File S2 Bivariate.pdf]

Statistical analysis of data included in Figure 11

Analysis of individual sensor detector change in white panel reflectance by temperature change in °C measured before and after field collections for 62 events across four years that show a loose relation. Data includes all three detectors of all sensors from the "Wolverine" field collections taken 2018-2021. Results produced using JMP, build 15.2.0 SAS Institute Inc. Cary North Carolina 27513.

Bivariate Fit of signal change (median_diff) by temperature change (temp_diff)

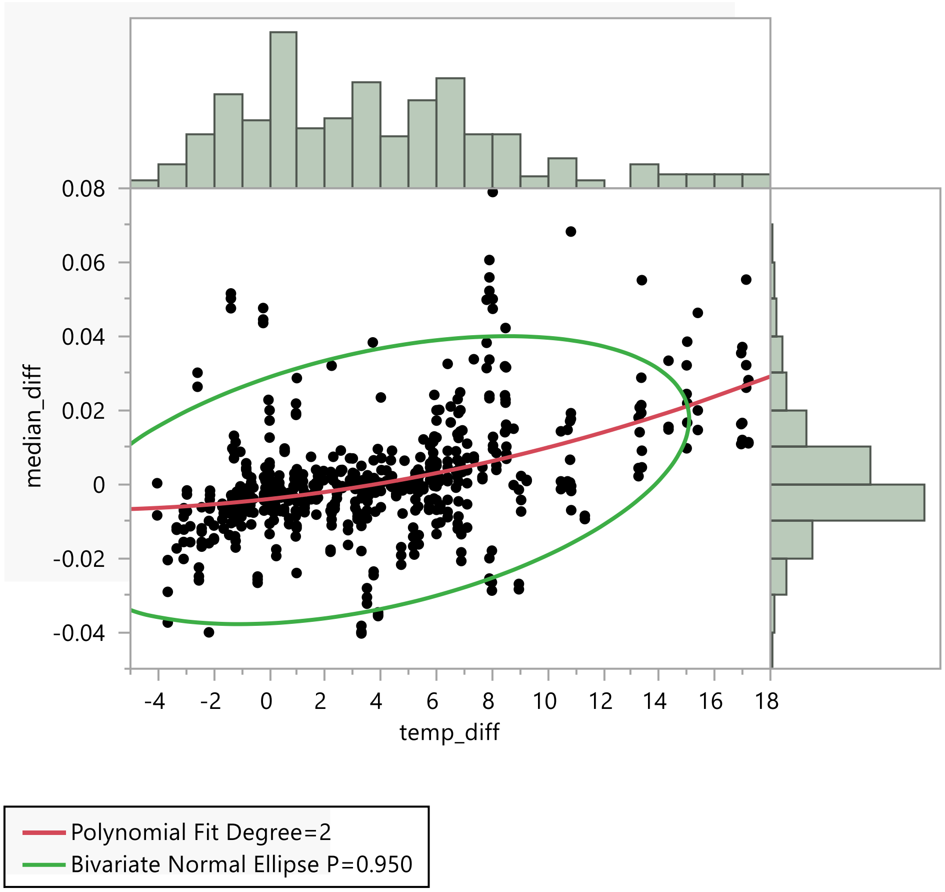

Summary Statistics

	Value	Lower 95%	Upper 95%	Signif. Prob
Correlation	0.42445	0.354497	0.489678	<.0001*
Covariance	0.03135			
Count	567			

Variable	Mean	Std Dev
temp_diff	3.723794	4.640122
median_diff	0.001031	0.015918

Polynomial Fit Degree=2

median_diff = -0.004824 + 0.0012478*temp_diff + 5.6213e-5*(temp_diff-3.72379)^2

Summary of Fit

RSquare	0.190149
RSquare Adj	0.187278
Root Mean Square Error	0.01435
Mean of Response	0.001031
Observations (or Sum Wgts)	567

Lack Of Fit

Source	DF	Sum of Squares	Mean Square	F Ratio
Lack Of Fit	161	0.07756890	0.000482	5.0339
Pure Error	403	0.03857121	0.000096	**Prob > F**
Total Error	564	0.11614011		<.0001*
				**Max RSq**
				0.7310

Analysis of Variance

Source	DF	Sum of Squares	Mean Square	F Ratio
Model	2	0.02726918	0.013635	66.2123
Error	564	0.11614011	0.000206	**Prob > F**
C. Total	566	0.14340929		<.0001*

Parameter Estimates

Term	Estimate	Std Error	t Ratio	Prob>|t|
Intercept	-0.004824	0.00079	-6.10	<.0001*
temp_diff	0.0012478	0.000152	8.20	<.0001*
(temp_diff-3.72379)^2	5.6213e-5	2.131e-5	2.64	0.0086*
