# Supplementary figures and images for: Proximal Active Optical Sensing Operational Improvement for Research Using the CropCircle ACS-470, Implications for Measurement of Normalized Difference Vegetation Index (NDVI)
Source: Sensors (Basel). 2023 May 24;23(11):5044. doi: 10.3390/s23115044 (PMC10255205; doi:10.3390/s23115044)

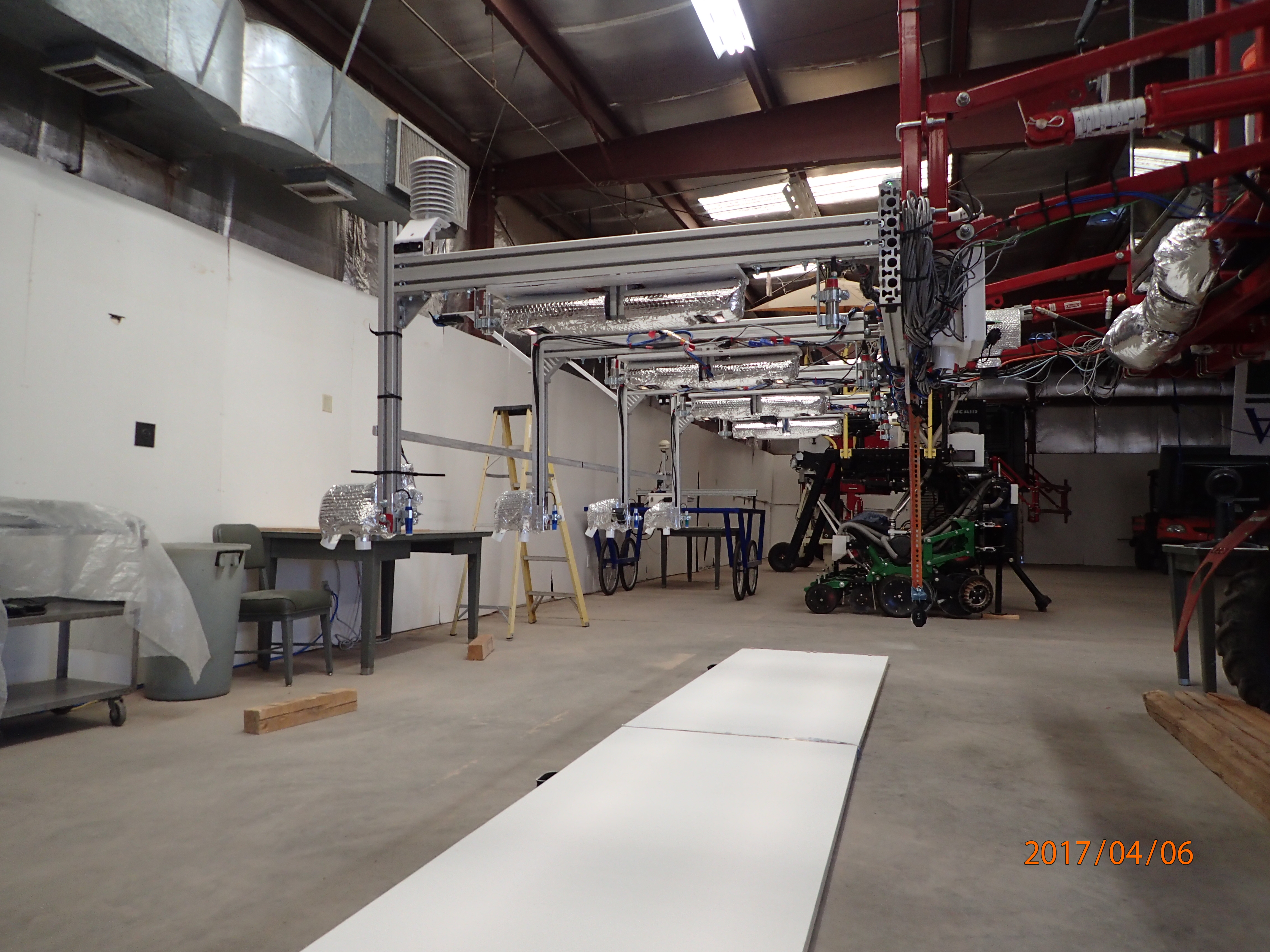

Supplement: Supplementary file 1 [file sensors-23-05044-s001.zip › Figure S1 Avenger rig.jpg]

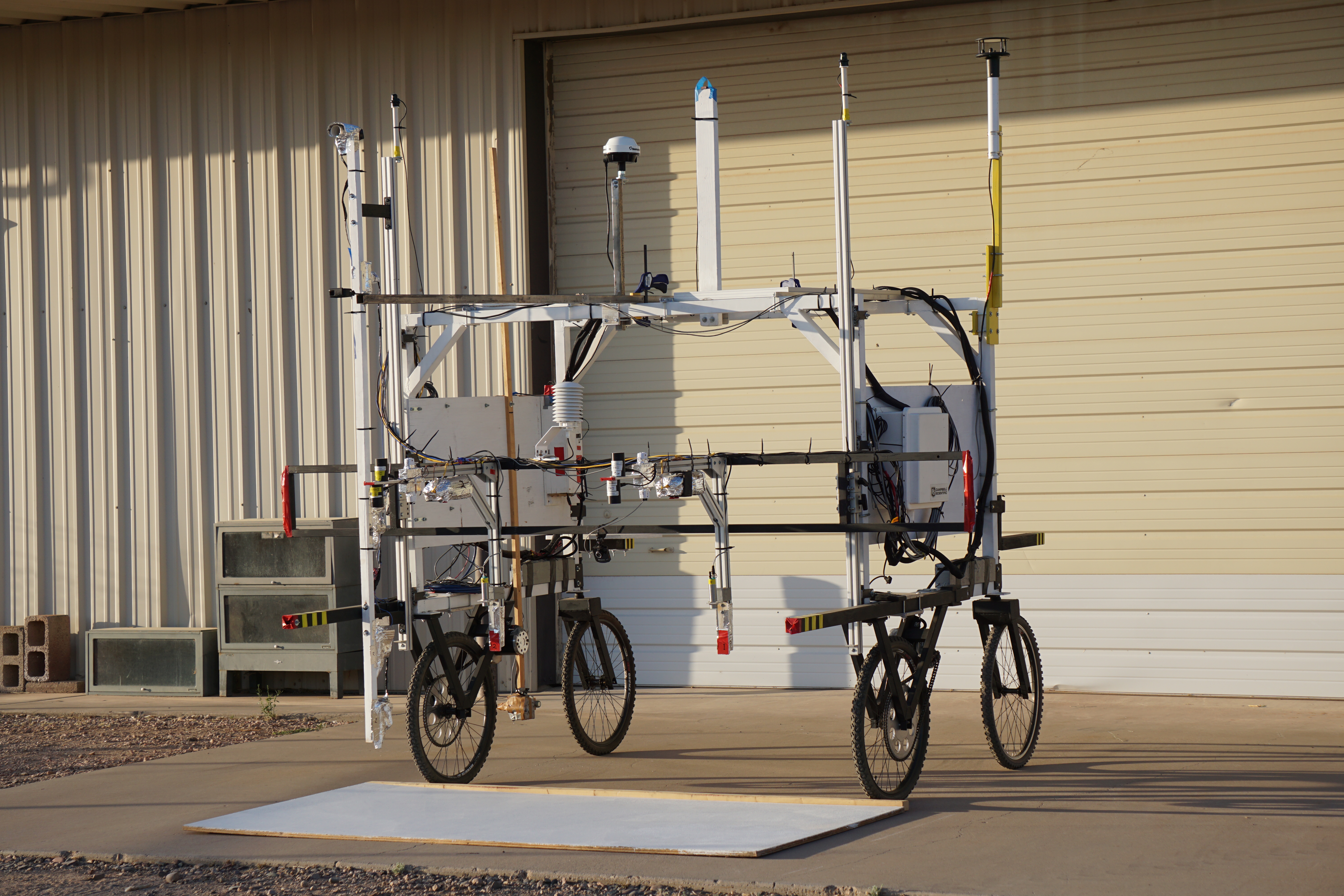

Supplement: Supplementary file 1 [file sensors-23-05044-s001.zip › Figure S2 Wolverine cart.jpg]

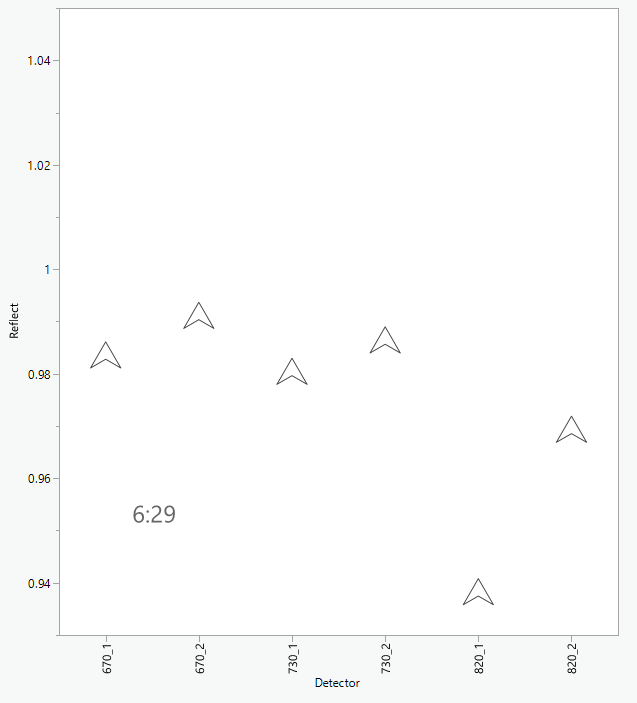

Supplement: Supplementary file 1 [file sensors-23-05044-s001.zip › Video S1 GIF warmup chart animation.gif]
